# Antihypertensive drugs use over a 5-year period among children and adolescents in Beijing, China: An observational study

**DOI:** 10.1097/MD.0000000000017411

**Published:** 2019-10-04

**Authors:** Yao Wu, Yaying Cao, Jing Song, Yaohua Tian, Mengying Wang, Man Li, Xiaowen Wang, Zhe Huang, Lin Li, Yaling Zhao, Xueying Qin, Yonghua Hu

**Affiliations:** a Department of Epidemiology and Biostatistics, School of Public Health, Peking University; b Department of Endocrinology, Chinese People's Liberation Army General Hospital, Beijing; c Department of Epidemiology and Biostatistics, School of Public Health, Xi’an Jiaotong University Health Science Center, Xi’an, Shaanxi, China.

**Keywords:** antihypertensive drugs, children and adolescents, hypertension

## Abstract

Evidence on the prescription patterns of antihypertensive drug use in children and adolescents in China is scarce. A descriptive analysis of the Beijing Medical Claim Data, which covered over 95% of the urban residents, was conducted to investigate antihypertensive prescribing patterns and trends in children and adolescents aged under 18 from 2009 to 2014 in Beijing, China. An additional meta-analysis of trends in hypertension prevalence was conducted to compare trends with antihypertensive medications.

A total of 11,882 patients received at least 1 prescription for antihypertensive drugs from 2009 to 2014. The number of annual antihypertensive users increased from 2009 to 2012, then declined steadily until 2014, which was consistent with the trend of the hypertension prevalence estimated from the meta-analysis. β-receptor blockers, thiazide diuretics, and angiotensin-converting enzyme inhibitors were the 3 most commonly prescribed antihypertensive drugs. More boys took the antihypertensive drugs than girls. For users aged under 3 years, thiazide diuretics, α-receptor blockers, and angiotensin-converting enzyme inhibitors were the most prescribed drugs, while β-receptor blockers, thiazide diuretics were the most used drugs for users above 3 years.

In conclusion, antihypertensive drug prescribing for children and adolescents increased from 2009 to 2014, with different characteristics in different subgroups.

## Abstract 2

Supplemental Digital Content is available in the text

## 1 Introduction

Hypertension is a worldwide chronic cardiovascular disease, which is the leading risk factor for disease burden^[1]^ and the major risk factor for cardiovascular disease.^[2]^ In recent years, hypertension has been increasingly recognized in pediatric populations due to an obesity epidemic.^[2,3]^ Blood pressure levels have increased in all age and sex groups among children, and the prevalence of hypertension was 14.5% in children and adolescents based on the National Student Health Study in 2010 in China.^[4]^ The China Health and Nutrition Survey found that the prevalence of hypertension increased substantially, from 8.06% in 1993 to 10.75% in 2011, among juveniles.^[5]^ Although early hypertension poses little immediate risk to most children, it carries the potential for future end-organ damage.^[3,6]^ Thus, pharmacological management is inevitably required, involving pharmacotherapy with mono- or combination therapy using thiazide diuretics, calcium channel blockers (CCBs), α-receptor blockers, β-receptor blockers, angiotensin-converting enzyme inhibitors (ACEIs), or angiotensin II receptor blockers (ARBs).

In 2017, 1 clinical practice guideline was issued by the American Academy of Pediatrics for screening and managing high blood pressure in children and adolescents.^[7]^ According to the guideline, pharmacologic treatment of hypertension should be initiated with ACEIs, ARBs, CCBs, or thiazide diuretics in children and adolescents,^[7,8]^ and β-receptor blockers are not recommended, which was inconsistent with guidelines in Europe^[9]^ and Canada.^[10]^ In China, the guidelines assume all major drug classes to be equivalent and suitable for initial or maintenance treatment for adults, but there are no guidelines developed specifically for the treatment of hypertension in children and adolescents,^[11]^ only listing several kinds of medications suitable for children and adolescents. We appreciate the viewpoint that there exist differences in pharmacology between adults and children,^[12]^ thus the application of guidelines of adults in children should be performed with caution. Meanwhile, little is known about the patterns in the prescription of antihypertensive drugs in children and adolescents in China.

The purpose of our study was to identify secular trends in the use of antihypertensive medications in children and adolescents in Beijing, China during a 5-year period (2009–2014), and to estimate prescription patterns of antihypertensive drugs in our study population with different demographic features.

## 2 Materials and methods

### 2.1 Data source

There are 3 main health insurance schemes in Beijing. For urban retirees and employees, there is the Urban Employee Basic Medical Insurance; the Urban Residence Basic Medical Insurance covers children, students, older adult people without previous employment, and unemployed people; and there is the New Rural Cooperative Medical Scheme for rural residents.

This descriptive study was performed using the Beijing Medical Claim Data from nationally funded basic health insurance that covers over 95% of all age residents in Beijing.^[13]^ The database contains basic demographics, prescribing information and medical treatment expenses. All drugs prescribed by doctors and dispensed by pharmacists were recorded in the database to track patient reimbursements. In this study, we extracted data for residents under the age of 18 years who were prescribed antihypertensive drugs, and we included the following variables: age, sex, levels of medical institutions, types of hospital visits, and antihypertensive drug information. However, we had no access to diagnostic information and information on clinical measurement and disease history. The data were accessible in our study from January 1st, 2009 to December 31st, 2014. The data were collected for administrative proposes and no individual names or identification numbers were used; therefore, no ethical approval was required for this study.

### 2.2 Measures

Antihypertensive drugs were categorized into 8 subclasses according to the Essential Medicine List, National Insurance Medicine List of China^[14]^ and Chinese National Formulary Chemicals and Biological Products for Children.^[15]^ These were β-receptor blockers, thiazide diuretics, α-receptor blockers, ACEIs, CCBs, ARBs, other vasodilators, and compound preparations (see Table S1, Supplemental Digital Content 1 which illustrates the list of all kinds of antihypertensive drugs doctors prescribed from 2009 to 2014). Users were divided into 5 age groups: infancy (aged 0–1), toddlerhood (aged 1–3), pre-school age (aged 3–6), school-age (aged 6–12), and adolescence (aged 12–18). According to the standard of management at different levels of hospitals, medical institutions were ranked into 3 levels: tertiary-level hospitals, secondary-level hospitals, and primary-level hospitals.^[16]^ Tertiary-level hospitals fall into 3 grades, which are tertiary grade A hospitals, tertiary grade B hospitals, and tertiary grade C hospitals. The types of hospital visits were defined as outpatient visits, inpatient visits, and emergency department visits. Hypertension may be treated with monotherapy, 2 drugs or fixed-dose combinations, and we divided the types of therapy into monotherapy and polytherapy (with ≥2 drugs or with fixed-dose combinations). The proportion of users receiving repeated prescriptions during our study period was calculated.

### 2.3 Statistical analysis

Descriptive analyses were performed to evaluate the sample characteristics over the study period. Frequency and proportions were calculated using units of users. To determine the frequency and proportions of users over the study period, each user was counted once. First, we presented yearly numbers and proportions of overall antihypertensive users. The numerator of the proportion was the number of drug users each year, and the denominator was the number of populations aged 0 to 19 years of the relevant years from 2009 to 2014 reported by Beijing Statistical Yearbooks.^[17–21]^ We used resident populations aged 0 to 19 years instead of resident populations aged 0 to 18, which were not calculated in the Beijing Statistical Yearbooks. Furthermore, because of the lack of specific numbers of populations aged under 19 in 2009, we multiply the whole population by the proportion of people aged under 19 years estimated by a sample survey to get the resident population aged under 19 years in 2009. Trends of the percentage of different antihypertensive classes from 2009 to 2014 were also presented. Then, we calculated the prescriptions and proportions of different antihypertensive classes according to the characteristics of age, sex, levels of medical institutions, types of hospital visits, and types of therapy. The statistical significance of subgroup differences was tested using the Chi-squared test.

We could not obtain the prevalence of hypertension directly from the database because we did not have access to diagnostic information, so a meta-analysis was conducted in our study to systematically review the studies reporting the prevalence of hypertension and to estimate the overall prevalence of hypertension among Chinese children and adolescents. Finally, the hypertension prevalence trends in this meta-analysis were compared with the trends in antihypertensive medication use estimated from the database. We briefly described the methods of meta-analysis: PubMed, Embase, the Chinese National Knowledge Infrastructure Database, and the Chinese Wanfang Database were independently searched by 2 reviewers for studies investigating the prevalence of hypertension among children and adolescents in China from inception to August 1, 2018. The following key terms “Hypertension,” “Blood pressure,” “Prevalence,” “Trend,” “Children,” “Child,” “Boys,” “Girls,” “Adolescents,” “China,” and “Chinese” were used in various combinations. The prevalence of hypertension was defined as the proportion of hypertension among all samples. The prevalence of hypertension was pooled according to the year, and an additional subgroup prevalence of sex was performed in each year. The methodology has been described in detail (see Paragraphs, Supplemental Digital Content 2, which describes the methodology of meta-analysis adopted in detail).

Microsoft SQL Server (version 2014) was used to extract data. All analyses were conducted in Stata 13.0 software (Copyright 1985–2013, StataCorp LP: College Station, Texas, USA).

## 3 Results

### 3.1 Baseline characteristics

The summary of the descriptive information of users recruited into this study is demonstrated in Table 1. A total of 11,882 users aged 0 to 18 years received at least 1 prescription for antihypertensive drugs from 2009 to 2014. Among them, the majority of users were aged 12 to 18 years (36.06%). More antihypertensive drugs were prescribed for males (58.06%) than females (41.94%). A total of 76.77% of users received antihypertensive drugs as inpatients, which is much more common than that for outpatients (20.19%) and emergency patients (3.04%). Tertiary level grade B hospitals accounted for 83.46% of the total users, much higher than tertiary-level grade A hospitals (16.40%). The proportion of users receiving repeated prescriptions during our study period was 23.03%.

**Table 1:**
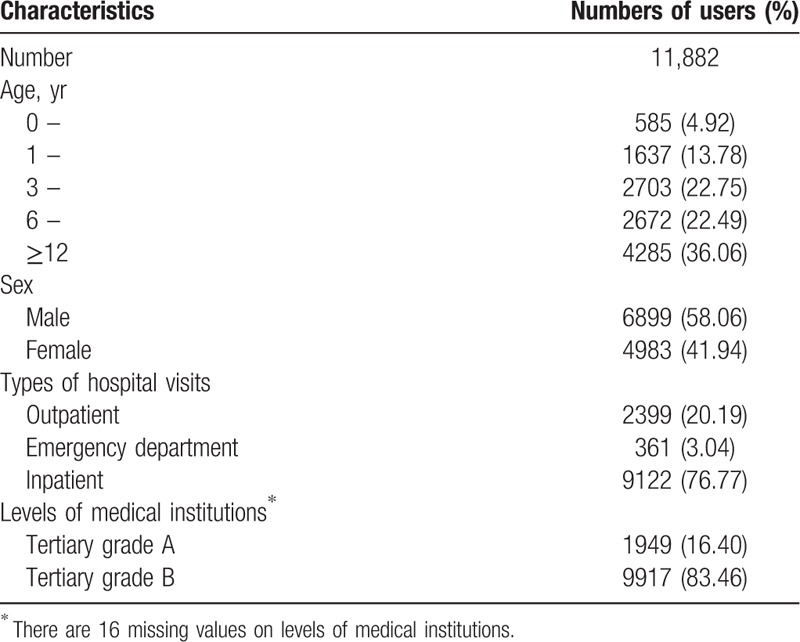
Characteristics of antihypertensive drug uses from 2009 to 2014 in children and adolescents in Beijing China.

### 3.2 Annual trend of antihypertensive drug users and drug usage prevalences

**Figure 1:**
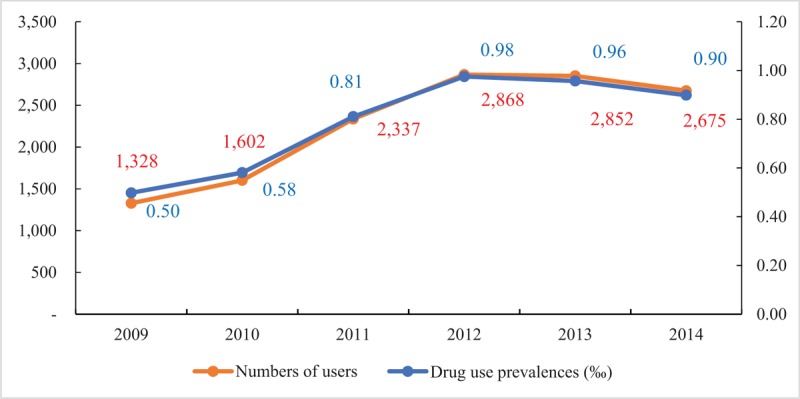
Annual trend of overall antihypertensive drug users and drug use prevalences in children and adolescents from 2009 to 2014 in Beijing, China. In the drug use prevalences, numerator is the number of users and denominator is local population aged 0 to 19 (2.76 million in 2009 and 2010, 2.88 million in 2011, 2.94 million in 2012, 2.98 million in 2013, and 2.98 million in 2014).

**Figure 2:**
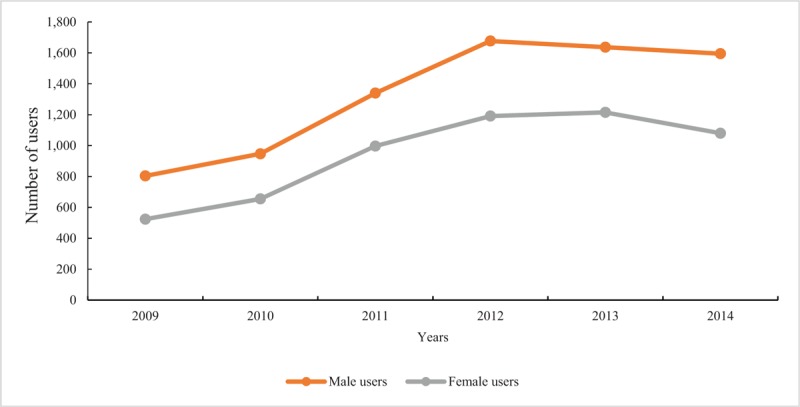
Numbers of users in different gender using antihypertensive drugs from 2009 to 2014.

Figure 1 shows the trends in the numbers of users aged 0 to 18 years who received antihypertensive drugs and drug-usage rates from 2009 to 2014. The total number of users in 2009 was 1328, which gradually increased to 2868 in 2012, and then began a slow decline to 2675 by 2014. This was a similar trend to the drug-usage rates. The trends were similar by sex, with more male users taking antihypertensive drugs than females in all study years (Fig. 2). For users aged above 6 years, total numbers of users increased annually while the numbers declined in users aged under 1 year, and remained almost stable in users aged 1 to 3 years. Furthermore, the numbers of users in each age group were similar before 2010 but demonstrated distinct differences in 2014. Users aged 12 to 18 years accounted for the largest proportion, followed by users aged 3 to 12 years and users aged 1 to 3 years, while users aged 0 to 1 year took up the smallest part (see Fig. S1, Supplemental Digital Content 3, which illustrates the numbers of users in different age using antihypertensive drugs from 2009 to 2014).

### 3.3 Prescription patterns

**Figure 3:**
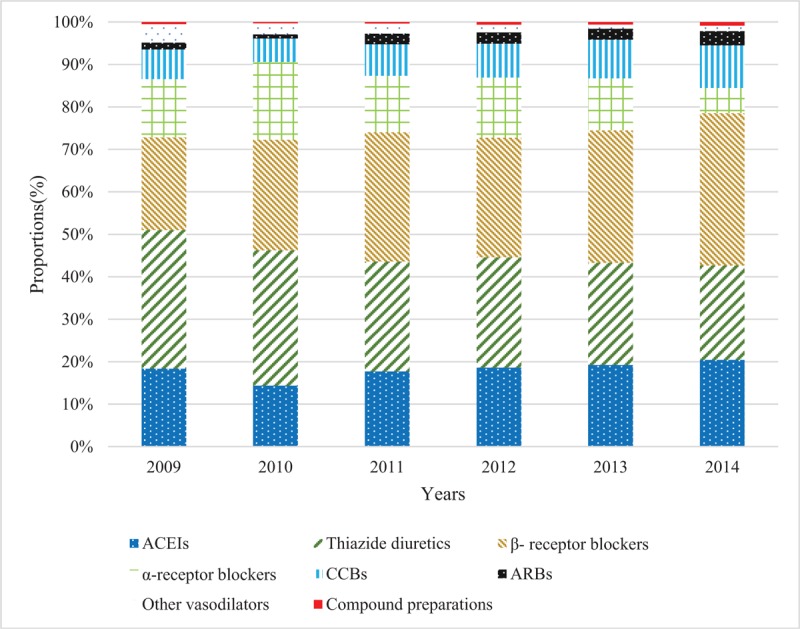
Proportions of users receiving different antihypertensive classes among children and adolescents from 2009 to 2014 in Beijing, China. ACEIs = angiotensin-converting enzyme inhibitors, CCBs = calcium channel blockers, ARBs = angiotensin II receptor blockers.

**Figure 4:**
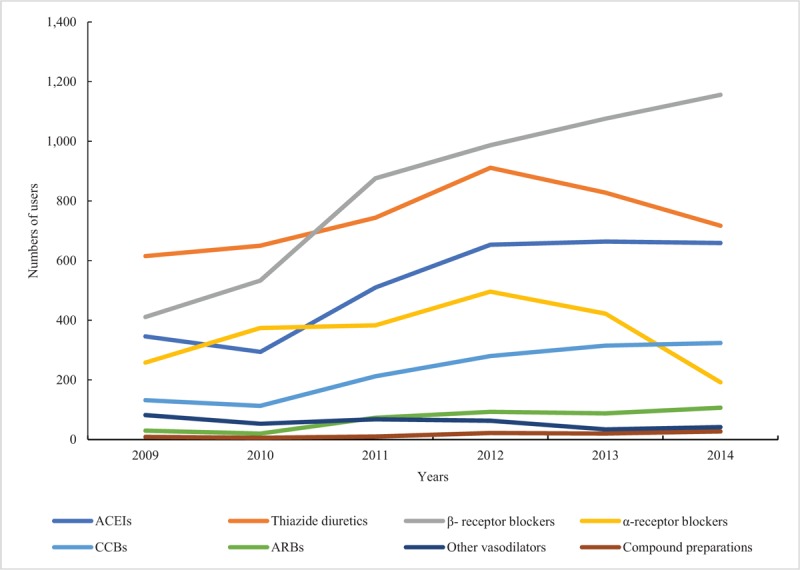
Total numbers of users receiving antihypertensive drugs from 2009 to 2014. ACEI = angiotensin-converting enzyme inhibitor, ARB = angiotensin receptor blocker, CCB = calcium channel blocker.

For both absolute numbers or proportions of drug users in different antihypertensive subclasses, thiazide diuretics, β-receptor blockers, and ACEIs were the most commonly prescribed antihypertensive drugs. Furthermore, the trends of prescribing β-receptor blockers, ACEIs, and CCBs increased from 2010 to 2014, while thiazide diuretics and α-receptor blockers started to decrease in 2012. Compound preparation, other vasodilators, and ARBs made up a smaller proportion of all drugs than other antihypertensive agents from 2009 to 2014, and their trends remained stable (Figs. 3 and 4).

**Figure 5:**
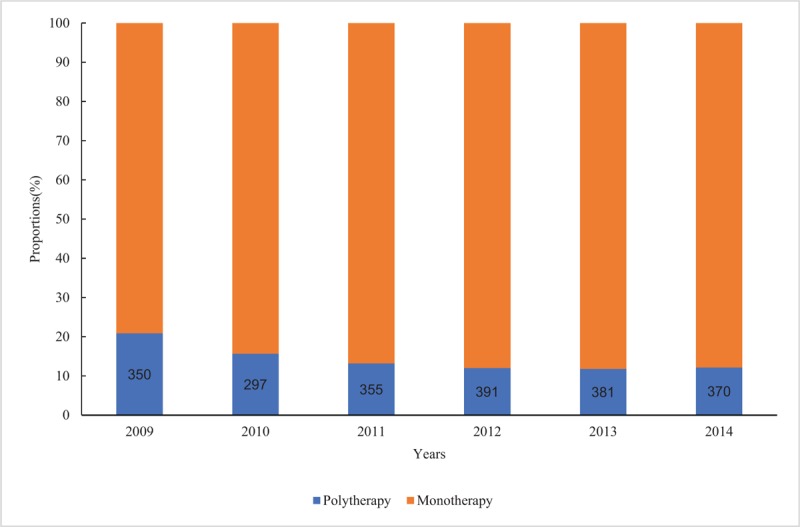
Antihypertensive drug use among children and adolescents by therapy types. Monotherapy indicates use of 1 active ingredient; polytherapy, use of >1 active ingredient.

Figure 5 shows user profiles in terms of monotherapy and polytherapy. The proportions of users receiving polytherapy decreased gradually from 2009 to 2011 and started to level off from 2012. But the total number of users receiving polytherapy (numbers in the blue bars) fluctuated a little from 350 in 2009 to 370 in 2014.

### 3.4 Characteristic-specific analysis of antihypertensive drug users

Table 2 shows the characteristic-specific prescribing patterns in antihypertensive drugs. Thiazide diuretics, ACEIs and α-receptor blocker were the most prescribed antihypertensive drugs in users aged 0 to 3 years (the proportions were 53.36%, 18.70%, and 15.74% in users aged 0–1 year, and 45.70%, 14.65%, and 26.04% in users aged 1–3 years, respectively). A total of 926 users (28.95%) aged 3 to 6 years were prescribed β-receptor blockers, making this the most prescribed drug in this age group, followed by thiazide diuretics (879, 27.48%), α-receptor blockers (747, 23.35%), and ACEIs (499, 15.60%). In users aged 6 to 18 years, β-receptor blockers, thiazide diuretics, and CCBs were the top 3 antihypertensive drugs, particularly in users aged 12 to 18 years (the proportions were 43.71%, 15.50%, and 14.62%, respectively). Male users aged under 18 were prescribed more drugs in each drug class than female users, and they had similar prescribing patterns. In terms of different types of hospital visits, most of the antihypertensive drugs were prescribed during inpatient and outpatient visits. ACEIs and β-receptor blockers were more likely to be prescribed in outpatient visits, the numbers of which were almost 2 times of the number of thiazide diuretics and CCBs. α-receptor blockers were the least prescribed drugs in outpatient visits. For inpatient visits, users of β-receptor blockers (3836, 31.67%) and thiazide diuretics (3345, 27.61%) accounted for a much greater share of total users, followed by α-receptor blockers (2038, 16.82%) and ACEIs (1692, 13.97%). Doctors prescribed more antihypertensive drugs of each subclass to patients in tertiary grade A hospitals than to patients in tertiary grade B hospitals, and the patterns were similar between tertiary grade A and grade B hospitals.

**Table 2:**
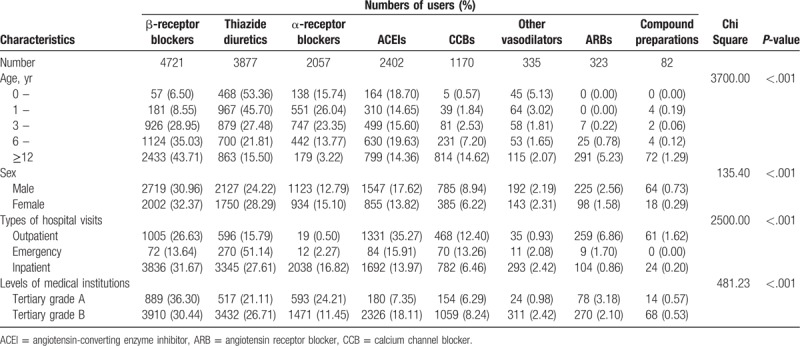
Characteristic-specific analysis of frequency and proportions of users aged 0 to 18 receiving antihypertensive drugs from 2009 to 2014 in Beijing, China.

### 3.5 Prevalence of hypertension among children and adolescents in China: a meta-analysis

**Figure 6:**
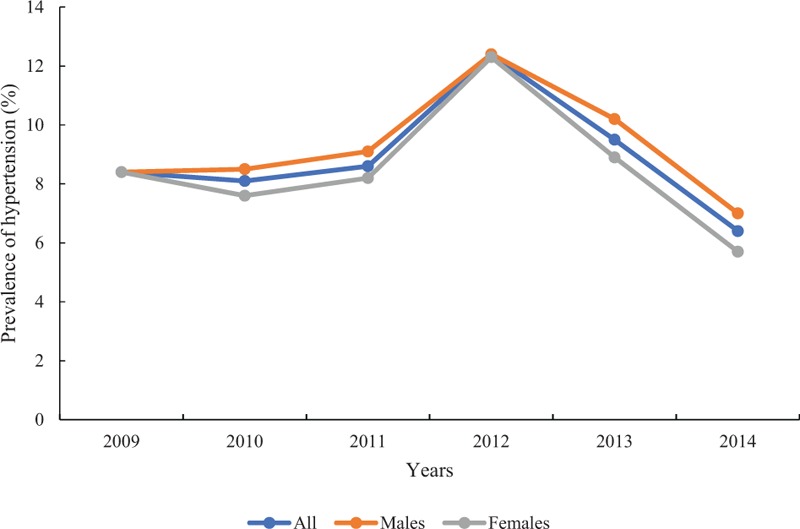
Pooled prevalence of hypertension among Chinese children and adolescents from 2009 to 2014.

The flowchart of the study search and selection procedure is attached in the supplementary file (see Fig. S2, Supplemental Digital Content 4, which illustrates the flowchart of study search and selection procedure in the meta-analysis). Nine studies indexed in databases were included in this meta-analysis (see Table S2, Supplemental Digital Content 5, which illustrates the characteristics of the studies included in the systematic review). The prevalence of hypertension gradually increased from 2009 (8.8%) to 2012 (12.4%) and then showed a marked decrease to 6.4% in 2014 (see Table S3, Supplemental Digital Content 6, which illustrates the overall and mean prevalence of hypertension among children and adolescents in subgroups by gender and year). In most years, males had a higher prevalence than females (Fig. 6).

## 4 Discussion

In this study, we found that there was a clear trend of increasing use of antihypertensive drugs over a 5-year period (2009–2014) in Beijing, China, particularly among users aged from 12 to 18 years. β-receptor blockers, thiazide diuretics, and ACEIs, were the most commonly prescribed antihypertensive drugs between 2009 and 2014. Antihypertensive medication uses present different characteristics in different subgroups. To the best of our knowledge, this is the first study to describe the long-term trend and patterns of antihypertensive prescribing among Chinese children and adolescents.

The numbers of antihypertensive users in our study increased from 2009 to 2012 and then decreased slightly until 2014, which was consistent with the trend in prevalence of hypertension over the same period in children and adolescents, as estimated from the meta-analysis. This demonstrated that the use of medications was closely related to the prevalence of hypertension. The reason for the prevalence of hypertension might be due to the very close association between hypertension and obesity in children and adolescents^[22,23]^ and the rising prevalence of obesity in this population in China.^[24]^ In the later age-specific analyses, we found that there were more adolescent antihypertensive drug users than there were children in younger age groups, and the gap became larger in recent years; the numbers of antihypertensive drug users increased over 5 years in the older age group, especially in 12 to 18 years age group, but decreased in the very young age group (age 0–1 year). The underlying explanation might be that the prevalence of hypertension was higher among adolescents than children aged under 12, and continued to increase in recent years^[25,26]^; therefore, antihypertensive drugs were more frequently prescribed to adolescents for hypertension therapy. In addition, previous studies showed the incidence rate of hyperthyroidism increased sharply with age, with most cases occurring during puberty,^[27,28]^ and β-receptor blockers were generally a routine therapeutic modality for the treatment of hyperthyroidism.^[29]^ That might partially explain the increasing number of β-receptor blockers prescriptions with age.

Our results showed that β-receptor blockers, thiazide diuretics, and ACEIs were the most commonly prescribed antihypertensive drugs among children and adolescents, and kept increasing throughout the study period; α-receptor blockers increased from 2009 to 2012, then declined until 2014; CCBs increased steadily over the 5 years although the total number was less than those of the 4 drugs mentioned above; ARBs, other vasodilators, and compound preparations were the least prescribed drugs, with numbers that remained stable from 2009 to 2014. Liberman et al^[30]^ found that β-receptor blockers had the highest prevalence among antihypertensive medications in US children and adolescents aged 6 to 18 years, followed by ACEIs, diuretics, CCBs, and ARBs, which was similar with our results.

In recent years, ACEIs have been shown to be commonly used as initial therapy for hypertension in the pediatric population.^[31]^ It is effective in reducing blood pressure and beneficial in protecting heart function, with few reported side effects.^[32]^ Dobson et al^[26]^ reported that the most commonly prescribed drug class was ACEIs, followed by β-receptor blockers, CCBs, and α-receptor blockers in US children aged 2 to 18 years. Yoon et al^[33]^ and Binka et al^[34]^ found that ACEIs were the most frequently prescribed monotherapy for adolescents in the US. These inconsistent results may be caused by inconsistency of guidelines, study periods, prices, and medication adherence in different regions.

As for the proportions of antihypertensive classes, we found an obvious growing trend of β-receptor blockers and a downward trend in thiazide diuretics. One reason for the changing proportions may be due to the change of age structures of drug users from 2009 to 2014 (see Fig. S1, Supplemental Content, which illustrates the numbers of users in different age using antihypertensive drugs from 2009 to 2014). The β-receptor blocker was the preferred medication for adolescents with hypertension because of its preventive effect on cardiovascular disease.^[35]^ Thus, the increasing number of adolescent users of antihypertensive drugs may be the cause of the rising proportion of β-receptor blockers. Meanwhile, the decline in the number of children aged under 1 year led to decreases in proportions of thiazide diuretics and α-receptor blockers, which were often used for acute heart failure treatment among patients at a very young age.^[36]^

For characteristic-specific analysis, we found that the antihypertensive prescription profiles also appeared to be different in different subgroups. Thiazide diuretics, α-receptor blockers, and vasodilator drugs were more prescribed in younger users, while β-receptor blockers, CCBs, and ARBs were more used in older groups. In very young age users, antihypertensive drugs might be more frequently prescribed for the treatment of acute and severe diseases; for example, thiazide diuretics, α-receptor blockers, and vasodilator drugs (such as sodium nitroprusside) are used for acute heart failure treatment.^[37,38]^ As mentioned above, β-receptor blockers were more used as a routine therapy for the treatment of hyperthyroidism in adolescents.^[29]^ These may explain the age differences in antihypertensive prescribing. Antihypertensive drugs were prescribed more commonly in males, which is consistent with previous findings^[33]^ and the results of the meta-analysis in our study. Moreover, we found that compared with outpatients, thiazide diuretics and α-receptor blockers accounted for a much greater share of total users in inpatient departments. To explain further, we conducted age-specific analyses among users of thiazide diuretics and α-receptor blockers. We found that among those who received α-receptor blockers and thiazide diuretics in inpatient departments, children aged under 6 accounted for a large part. One reason may be that children and adolescents admitted to the hospital tended to be in more serious condition, and the American Academy of Pediatrics guideline suggested that if ACEIs, ARBs, CCBs, or thiazide diuretics failed to achieve adequate blood pressure control, other antihypertensive medications like α-receptor blockers should be prescribed.^[7]^

In this study, we also found that the use of combination therapy was low as compared with monotherapy. The proportion of combination therapy declined slowly with the increasing use of antihypertensive drugs. Combination therapy was the major treatment for hypertension in adults; a previous study showed that the proportion of prescriptions involving combination therapy was larger than that of monotherapy when treating hypertension in Chinese adults.^[39]^ Considering the lack of a medication guideline for hypertension among children and adolescents, when treating juveniles, some doctors may use the same method used for treating adults. In addition, polytherapy may also be considered as an initial treatment of severe hypertension or other cardiovascular events.^[40]^ Compared with the studies among adults, which described a declining trend in the percentage of polytherapy,^[36]^ our results demonstrated that medicines among children and adolescents were prescribed cautiously. This might be to minimize adverse drug reactions caused by polytherapy.^[41]^

This study has several limitations. First, we had no access to diagnostic information; therefore, we could not clarify whether the antihypertensive drugs were prescribed for the treatment of hypertension and we may overestimate the use of antihypertensive drugs for hypertension. Second, the meta-analysis in our study demonstrated high percentages of variation attributable to heterogeneity, which reduce the accuracy of the estimated prevalence. But our aim was to compare the trend in the prevalence of hypertension with the trend in the number of antihypertensive users. The results of our meta-analysis could still serve as a reference. Third, when calculating the yearly proportions of overall antihypertensive users, we used the number of populations aged 0 to 19 years from the Beijing Statistical Yearbook as the denominator, which underestimates the drug-usage prevalence.

In the future research, we will seek to obtain diagnostic information of the database to further analyze the antihypertensive drug compliance and prescription rationality in this Chinese population, and further extend the analysis year to 10 years or even longer, and explore the long-term trends and characteristics of antihypertensive drug prescribing changes.

In conclusion, our study demonstrated antihypertensive prescription profiles among children and adolescents in Beijing, China. There was an increase in antihypertensive drug prescribing for children and adolescents from 2009 to 2014. Antihypertensive medication uses present different characteristics in different subgroups. We propose more detailed guidelines for prescribing hypertension medications among Chinese children and adolescents, and more research is needed to ascertain the compliance with guidelines among children and adolescents.

## Author contributions

**Conceptualization:** Xueying Qin.

**Data curation:** Yaohua Tian, Mengying Wang.

**Formal analysis:** Yao Wu, Yaying Cao, Jing Song.

**Funding acquisition:** Xueying Qin, Yonghua Hu.

**Methodology:** Yao Wu.

**Project administration:** Yonghua Hu.

**Software:** Yao Wu.

**Supervision:** Xueying Qin, Yonghua Hu.

**Writing – original draft:** Yao Wu.

**Writing – review & editing:** Man Li, Xiaowen Wang, Zhe Huang, Lin Li, Yaling Zhao, Xueying Qin.

## Supplementary Material

Supplemental Digital Content
