# Supplementary material for: Antihypertensive drugs use over a 5-year period among children and adolescents in Beijing, China: An observational study
Source: Medicine (Baltimore). 2019 Oct 4;98(40):e17411. doi: 10.1097/MD.0000000000017411 (PMC6783152; doi:10.1097/MD.0000000000017411)
Supplement: Supplemental Digital Content [file medi-98-e17411-s001.doc]

**Supplemental Digital Content 1. Table S1** that illustrates the list of all kinds of antihypertensive drugs doctors prescribed from 2009 to 2014. docx

**Antihypertensive Drugs Use over a 5-year period among Children and Adolescents in Beijing, China**

Yao Wu,BS^1^, Yaying Cao, PhD^1^, Jing Song, PhD^1^, Yaohua Tian, PhD^1^, Mengying Wang, MS^1^, Man Li, MS^1^, Xiaowen Wang, BS^1^, Zhe Huang, MS^1^, Lin Li, MD^2^, Yaling Zhao, PhD^3^, Xueying Qin, PhD^1^ & Yonghua Hu, MD^1^

^1^ Department of Epidemiology and Biostatistics, School of Public Health, Peking University, 100191, Beijing, China, ^2^ Department of Endocrinology, Chinese People’s Liberation Army General Hospital, 100088, Beijing, China, ^3^ Department of Epidemiology and Biostatistics, School of Public Health, Xi’an Jiaotong University Health Science Center, 710061, Xi’an, Shaanxi, China

Xueying Qin and Yonghua Hu jointly supervised this work. Correspondence and requests for materials should be addressed to Xueying Qin (email: xueyingqin@bjmu.edu.cn) or Yonghua Hu (email: yhhu@bjmu.edu.cn)

**Table S1. List of all kinds of antihypertensive drugs doctors prescribed from 2009 to 2014.**

| **Calcium Channel Blocker** | **Compound preparations** | **ACEIs** | **β-receptor blockers** | **ARBs** | **Thiazide diuretics** | **α-receptor blockers** | **Other vasodilators** |
| --- | --- | --- | --- | --- | --- | --- | --- |
| Diltiazem | Valsartan and hydrochlorothiazide | Enalapril | Carvedilol | Telmisartan | Indapamide | Phentolamine | Sodium Nitroprusside |
| Amlodipine | Perindopril and Indapamide | Lisinopril | Sotalol | Irbesartan | Hydrochlorothiazide | Urapidil | Niacin |
| Nifedipine | Losartan Potassium and Hydrochlorothiazide | Captopril | Propranolol | Valsartan | Bumetanide | Prazosin | Papaverine |
| Nicardipine | Irbesartan and Hydrochlorothiazide | Benazepril | Bisoprolol | Candesartan | Torasemide | Phenoxybenzamine | Dibazole |
| Nimodipine | Compound Amiloride Hydrochloride | Ramipril | Metoprolol | Losartan Potassium | Amiloride |  |  |
| Verapamil | Compound Reserpine | Perindopril | Atenolol | Olmesartan | Furosemide |  |  |
| Felodipine | Valsartan Amlodipine | Fosinopril | Labetalol |  | Spironolactone |  |  |
| Lacidipine | Enalapril Maleate | Imidapril | Arotinolol |  |  |  |  |
| Benidipine |  |  | Esmolol |  |  |  |  |
| Levamlodipine |  |  |  |  |  |  |  |
| Nitrendipine |  |  |  |  |  |  |  |
| Lercanidipine |  |  |  |  |  |  |  |

**Supplemental Digital Content 5. Table S2** that illustrates the characteristics of the studies included in the systematic review. docx

**Antihypertensive Drugs Use over a 5-year period among Children and Adolescents in Beijing, China**

Yao Wu,BS^1^, Yaying Cao, PhD^1^, Jing Song, PhD^1^, Yaohua Tian, PhD^1^, Mengying Wang, MS^1^, Man Li, MS^1^, Xiaowen Wang, BS^1^, Zhe Huang, MS^1^, Lin Li, MD^2^, Yaling Zhao, PhD^3^, Xueying Qin, PhD^1^ & Yonghua Hu, MD^1^

^1^ Department of Epidemiology and Biostatistics, School of Public Health, Peking University, 100191, Beijing, China, ^2^ Department of Endocrinology, Chinese People’s Liberation Army General Hospital, 100088, Beijing, China, ^3^ Department of Epidemiology and Biostatistics, School of Public Health, Xi’an Jiaotong University Health Science Center, 710061, Xi’an, Shaanxi, China

Xueying Qin and Yonghua Hu jointly supervised this work. Correspondence and requests for materials should be addressed to Xueying Qin (email: xueyingqin@bjmu.edu.cn) or Yonghua Hu (email: yhhu@bjmu.edu.cn)

**Table S2. Characteristics of the studies included in the mata-analysis.**

| **First author** | **Year published** | **Study Period** | **Location** | **Sample population** | **Event counts** | **Prevalence** | **Gender** | **Age** |
| --- | --- | --- | --- | --- | --- | --- | --- | --- |
| Tao Xu^1^ | 2015 | 2007-2011 | Jiangsu, Shandong, Henan, Hubei,  Human, Guangxi, and Guizhou | 14193 | 672 | 4.70 | Male | 8-18 |
|  |  |  |  | 15804 | 572 | 3.60 | Female |  |
|  |  |  |  | 29997 | 1244 | 4.15 | Overall |  |
| Bin Dong^2^ | 2015 | 2010 | 31 provinces | 98606 | 6687 | 6.80 | Male | 7-17 |
|  |  |  |  | 98585 | 5729 | 5.80 | Female |  |
|  |  |  |  | 197191 | 12416 | 6.30 | Overall |  |
| Weili Yan^3^ | 2016 | 2009 | Jiangsu, Shandong, Henan, Hubei,  Human, Guangxi, and Guizhou | 569 | 61 | 11.80 | Male | 7-17 |
|  |  | 2011 |  | 658 | 78 | 12.34 | Male |  |
|  |  | 2009 |  | 458 | 62 | 14.69 | Female |  |
|  |  | 2011 |  | 639 | 56 | 9.12 | Female |  |
|  |  | 2009 |  | 1027 | 123 | 13.10 | Overall |  |
|  |  | 2011 |  | 1297 | 134 | 10.75 | Overall |  |
| Yide Yang^4^ | 2017 | 2013 | Liaoning, Tianjin, Ningxia, Shanghai, Chongqing, Hunan and Guangdong | 62168 | 5933 | 9.50 | Overall | 6-17 |
|  |  |  |  | 32064 | 3262 | 10.20 | Male |  |
|  |  |  |  | 30104 | 2671 | 8.90 | Female |  |
| Zhiyong Zou^5^ | 2017 | 2014 | 31 provinces | 106982 | 7489 | 7.00 | Male | 7-18 |
|  |  |  |  | 106979 | 6098 | 5.70 | Female |  |
|  |  |  |  | 214354 | 13719 | 6.40 | Overall |  |
| Yanhui Dong^6^ | 2018 | 2013 | Liaoning, Tianjin, Ningxia, Shanghai, Chongqing,  Hunan, Guangdong | 25558 | 2564 | 10.03 | Male | 6-17 |
|  |  |  |  | 24778 | 2166 | 8.74 | Female |  |
|  |  |  |  | 50336 | 4722 | 9.38 | Overall |  |
| Dongmei Yu^7^ | 2018 | 2010-2012 | Jiangsu, Shandong, Henan, Hubei,  Human, Guangxi, and Guizhou | 18032 | 2236 | 12.40 | Male | 6-17 |
|  |  |  |  | 17625 | 2168 | 12.30 | Female |  |
|  |  |  |  | 35657 | 4421 | 12.40 | Overall |  |

**Reference:**

1. Xu T, Zhu G, Liu J, Han S. Gender-specific prevalence and associated risk factors of high normal blood pressure and hypertension among multi-ethnic Chinese adolescents aged 8–18 years old. *Blood pressure.* 2015;24(3):189-195.

2. Dong B, Wang Z, Wang H-J, Ma J. Population attributable risk of overweight and obesity for high blood pressure in Chinese children. *Blood pressure.* 2015;24(4):230-236.

3. Yan WL, Li XS, Zhang Y, et al. Reevaluate secular trends of body size measurements and prevalence of hypertension among Chinese children and adolescents in past two decades. *Journal of hypertension.* 2016;34(12):2337-2343.

4. Yang YD, Dong B, Wang S, et al. Prevalence of high blood pressure subtypes and its associations with BMI in Chinese children: a national cross-sectional survey. *BMC public health.* 2017;17(1):598-605.

5. Zou ZY, Dong YH, Ma J. The endemic distribution and related factors of elevated blood pressure among Chinese children and adolescents aged 7-18 years in 2014. *Chinese Journal of Preventive Medicine.* 2017;51(4):290-294.

6. Dong Y, Song Y, Zou Z, Ma J, Dong B, Prochaska JJ. Updates to pediatric hypertension guidelines: influence on classification of high blood pressure in children and adolescents. 2018:297-306.

7. Yu DM, Xu XL, Gao X, et al. Status of blood pressure and prevalence of hypertension among 6-17 years old children and adolescents in 2010-2012 in China. *Journal of Hygiene Research.* 2018;47(1):1-6.

**Supplemental Digital Content 6. Table S3** that illustrates the overall and mean prevalence of hypertension among children and adolescents in subgroups by gender and year. docx

**Antihypertensive Drugs Use over a 5-year period among Children and Adolescents in Beijing, China**

Yao Wu,BS^1^, Yaying Cao, PhD^1^, Jing Song, PhD^1^, Yaohua Tian, PhD^1^, Mengying Wang, MS^1^, Man Li, MS^1^, Xiaowen Wang, BS^1^, Zhe Huang, MS^1^, Lin Li, MD^2^, Yaling Zhao, PhD^3^, Xueying Qin, PhD^1^ & Yonghua Hu, MD^1^

^1^ Department of Epidemiology and Biostatistics, School of Public Health, Peking University, 100191, Beijing, China, ^2^ Department of Endocrinology, Chinese People’s Liberation Army General Hospital, 100088, Beijing, China, ^3^ Department of Epidemiology and Biostatistics, School of Public Health, Xi’an Jiaotong University Health Science Center, 710061, Xi’an, Shaanxi, China

Xueying Qin and Yonghua Hu jointly supervised this work. Correspondence and requests for materials should be addressed to Xueying Qin (email: xueyingqin@bjmu.edu.cn) or Yonghua Hu (email: yhhu@bjmu.edu.cn)

**Table S3. Overall and mean prevalence of hypertension among children and adolescents in subgroups by gender and year.**

| **Items** | | **Prevalence (%)** | **95% Confidence intervals (CI)** | ***I^2*^*** |
| --- | --- | --- | --- | --- |
| 2009 | Overall | 8.8 | 4.5-13.1 | 99.5% |
|  | Male | 8.7 | 4.4-13.1 | 99.9% |
|  | Female | 8.7 | 4.4-13.0 | 99.6% |
| 2010 | Overall | 7.7 | 5.4-10.1 | 99.9% |
|  | Male | 8.0 | 5.8-10.3 | 99.7% |
|  | Female | 7.4 | 5.2-9.6 | 99.7% |
| 2011 | Overall | 8.1 | 4.3-11.9 | 99.9% |
|  | Male | 8.4 | 4.9-11.8 | 99.7% |
|  | Female | 7.8 | 4.3-11.3 | 99.8% |
| 2012 | Overall | 9.4 | 3.5-15.3 | - |
|  | Male | 9.4 | 3.4-15.3 | - |
|  | Female | 9.4 | 3.9-15.0 | - |
| 2013 | Overall | 9.5 | 4.8-23.9 | - |
|  | Male | 10.2 | 9.9-10.5 | - |
|  | Female | 8.9 | 8.6-9.2 | - |
| 2014 | Overall | 6.4 | 6.3-6.5 | - |
|  | Male | 7.0 | 6.8-7.2 | - |
|  | Female | 5.7 | 5.6-5.8 | - |

* For the year2012-2014, I^2^ was not applicable because only one study was included.

**Supplemental Digital Content 2. Paragraphs** that describes the methodology of Meta-analysis adopted in detail. docx

**Antihypertensive Drugs Use over a 5-year period among Children and Adolescents in Beijing, China**

Yao Wu,BS^1^, Yaying Cao, PhD^1^, Jing Song, PhD^1^, Yaohua Tian, PhD^1^, Mengying Wang, MS^1^, Man Li, MS^1^, Xiaowen Wang, BS^1^, Zhe Huang, MS^1^, Lin Li, MD^2^, Yaling Zhao, PhD^3^, Xueying Qin, PhD^1^ & Yonghua Hu, MD^1^

^1^ Department of Epidemiology and Biostatistics, School of Public Health, Peking University, 100191, Beijing, China, ^2^ Department of Endocrinology, Chinese People’s Liberation Army General Hospital, 100088, Beijing, China, ^3^ Department of Epidemiology and Biostatistics, School of Public Health, Xi’an Jiaotong University Health Science Center, 710061, Xi’an, Shaanxi, China

Xueying Qin and Yonghua Hu jointly supervised this work. Correspondence and requests for materials should be addressed to Xueying Qin (email: xueyingqin@bjmu.edu.cn) or Yonghua Hu (email: yhhu@bjmu.edu.cn)

**The methodology of Meta-analysis adopted in detail**

**Data selection**

Pubmed, Embase, Chinese National Knowledge Infrastructure database and Chinese Wanfang database were independently searched by two reviewers for studies investigating the prevalence of hypertension among children and adolescents in China from inception to August 1, 2018. The following key terms “Hypertension”, “Blood pressure”, “Prevalence”, “Trend”, “Children”, “Child”, “Boys”, “Girls”, “Adolescents”, “China” and “Chinese” were used in various combinations. Prevalence was calculated for each year.

Two reviewers screened all identified articles for inclusion based on the following inclusion criteria: (1) cross-sectional epidemiological studies that provided the prevalence of hypertension among children and adolescents in China; (2) nationwide studies; (3) studies with a full text; (4) patients diagnosed using validated diagnostic criteria. Studies met any of following characteristics were excluded: (1) studies based on data included via another study; (2) study period was not clearly defined; (3) reviews, opinions, editorials, letters, reports and commentaries.

**Data extraction**

Data extraction was conducted independently by two authors and any disagreement between two authors was resolved by the third reviewer. The following information were extracted using a standardized form: name of the first author, year of publication, date of investigation, sample population, event counts, gender and prevalence of hypertension.

**Statistical analysis**

In this study, the prevalence of hypertension was defined as the proportion of hypertension among all samples. We assessed heterogeneity by using *I^2^* statistic, which indicated the percentage of variation attributable to heterogeneity. If *I^2^* was below 50%, we chose the fixed-effects model because of a low heterogeneity, while if *I^2^* was above 50% or 75%, which indicated moderate or high heterogeneity, respectively, we chose the random model. The STATA 13.0 software (Copyright 1985-2013 Stata Corp LP) was used to calculate the pooled prevalence of hypertension and those of subgroup based on gender.

**Supplemental Digital Content 3. Figure S1** that illustrates the numbers of users in different age using antihypertensive drugs from 2009 to 2014. Note: ``1 to 3'' means greater than 1 and less than or equal to 3, and so on. docx

**Antihypertensive Drugs Use over a 5-year period among Children and Adolescents in Beijing, China**

Yao Wu,BS^1^, Yaying Cao, PhD^1^, Jing Song, PhD^1^, Yaohua Tian, PhD^1^, Mengying Wang, MS^1^, Man Li, MS^1^, Xiaowen Wang, BS^1^, Zhe Huang, MS^1^, Lin Li, MD^2^, Yaling Zhao, PhD^3^, Xueying Qin, PhD^1^ & Yonghua Hu, MD^1^

^1^ Department of Epidemiology and Biostatistics, School of Public Health, Peking University, 100191, Beijing, China, ^2^ Department of Endocrinology, Chinese People’s Liberation Army General Hospital, 100088, Beijing, China, ^3^ Department of Epidemiology and Biostatistics, School of Public Health, Xi’an Jiaotong University Health Science Center, 710061, Xi’an, Shaanxi, China

Xueying Qin and Yonghua Hu jointly supervised this work. Correspondence and requests for materials should be addressed to Xueying Qin (email: xueyingqin@bjmu.edu.cn) or Yonghua Hu (email: yhhu@bjmu.edu.cn)


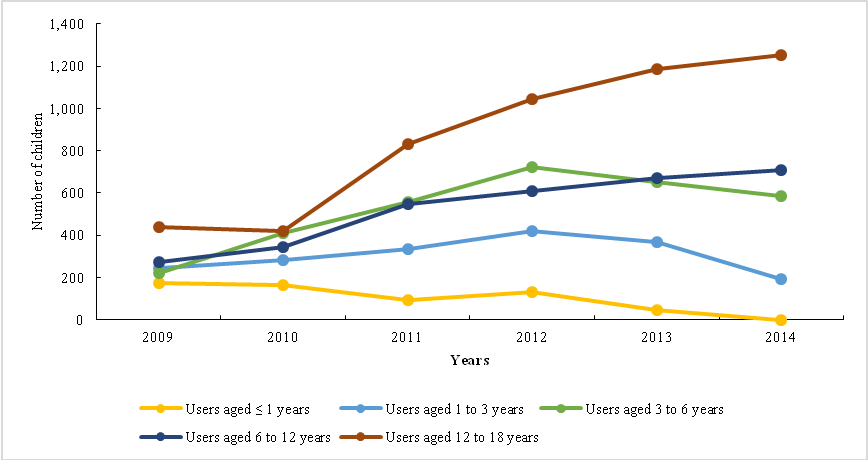


**Figure S1.** Numbers of users in different age using antihypertensive drugs from 2009 to 2014. Note: “1 to 3” means greater than 1 and less than or equal to 3, and so on.

**Supplemental Digital Content 4. Figure S2** that illustrates the flowchart of study search and selection procedure. docx

**Antihypertensive Drugs Use over a 5-year period among Children and Adolescents in Beijing, China**

Yao Wu,BS^1^, Yaying Cao, PhD^1^, Jing Song, PhD^1^, Yaohua Tian, PhD^1^, Mengying Wang, MS^1^, Man Li, MS^1^, Xiaowen Wang, BS^1^, Zhe Huang, MS^1^, Lin Li, MD^2^, Yaling Zhao, PhD^3^, Xueying Qin, PhD^1^ & Yonghua Hu, MD^1^

^1^ Department of Epidemiology and Biostatistics, School of Public Health, Peking University, 100191, Beijing, China, ^2^ Department of Endocrinology, Chinese People’s Liberation Army General Hospital, 100088, Beijing, China, ^3^ Department of Epidemiology and Biostatistics, School of Public Health, Xi’an Jiaotong University Health Science Center, 710061, Xi’an, Shaanxi, China

Xueying Qin and Yonghua Hu jointly supervised this work. Correspondence and requests for materials should be addressed to Xueying Qin (email: xueyingqin@bjmu.edu.cn) or Yonghua Hu (email: yhhu@bjmu.edu.cn)

**Figure S2.** The flowchart of study search and selection procedure in the meta-analysis.
